# Characterization of a Family of Novel Cysteine- Serine-Rich Nuclear Proteins (CSRNP)

**DOI:** 10.1371/journal.pone.0000808

**Published:** 2007-08-29

**Authors:** Sébastien Gingras, Stéphane Pelletier, Kelli Boyd, James N. Ihle

**Affiliations:** 1 Department of Biochemistry, St. Jude Children's Research Hospital, Memphis, Tennessee, United States of America; 2 Animal Resource Center, St. Jude Children's Research Hospital, Memphis, Tennessee, United States of America; University of Minnesota, United States of America

## Abstract

Gene array analysis has been widely used to identify genes induced during T cell activation. Our studies identified an immediate early gene that is strongly induced in response to IL-2 in mouse T cells which we named cysteine- serine-rich nuclear protein-1 (CSRNP-1). The human ortholog was previously identified as an AXIN1 induced gene (AXUD1). The protein does not contain sequence defined domains or motifs annotated in public databases, however the gene is a member of a family of three mammalian genes that share conserved regions, including cysteine- and serine-rich regions and a basic domain, they encode nuclear proteins, possess transcriptional activation domain and bind the sequence AGAGTG. Consequently we propose the nomenclature of CSRNP-1, -2 and -3 for the family. To elucidate the physiological functions of CSRNP-1, -2 and -3, we generated mice deficient for each of these genes by homologous recombination in embryonic stem cells. Although the CSRNP proteins have the hallmark of transcription factors and CSRNP-1 expression is highly induced by IL-2, deletion of the individual genes had no obvious consequences on normal mouse development, hematopoiesis or T cell functions. However, combined deficiencies cause partial neonatal lethality suggesting that the genes have redundant functions.

## Introduction

The differentiation and functions of T cells are regulated by many cytokines, including the family of interleukins (IL) sharing the common cytokine receptor γ chain (IL-2, IL-4, IL-7, IL-9, IL-15, and IL-21). IL-2 plays an essential role in development of peripheral T cell populations by promoting cell survival and driving expansion of antigens-stimulated T cells. It also stimulates proliferation and differentiation of B cells and augments the cytotoxic activity of NK cells [1], [2]. IL-2 is also required for the generation of a unique population of CD4+CD25+ regulatory T cells in the thymus [3]–[5]. Lastly, IL-2 has been proposed to regulate clonal contraction by sensitizing activated T cells to undergo activation-induced cell death (AICD) upon TCR re-stimulation [6]–[9].

Although the physiological importance of IL-2 signaling is established from the above studies, many of the IL-2 signaling events that mediate the responses are unknown. However, the derivation of Stat5a/b deficient mice demonstrated that this transcription factor plays a central role in the response of peripheral T cells to IL-2 [10], [11] and thus implicating gene regulation as a critical event. [12]–[15]. These studies have identified a number of genes that are induced by IL-2. For example, our previous studies have identified genes such as cytokine inducible SH2 containing protein (CIS), a member of the SOCS gene family, the cytokine Oncostatin M (OSM) and GADD45γ. The physiological relevance of these target genes has been explored by deriving mice in which the genes for CIS (unpublished data), GADD45γ [16] and OSM [17] were deleted and somewhat remarkably none of the deletions had significant consequences to T cell development or function.

Here, we report on the identification of a novel gene induced by IL-2 in mouse T cells that we named cysteine- serine-rich nuclear protein-1 (CSRNP-1). During the course of our studies, the human orthologue of the gene, AXUD1, was identified in an array analysis of genes induced in a colon cancer cell line by over-expression of AXIN1 [18]. As detailed here, CSRNP-1 is a member of a novel family of genes encoding nuclear proteins that contain cysteine- and serine-rich domains. Consequently, we propose the nomenclature CSRNP-1, -2 and -3 for the family. To explore the physiological functions of the family members, we generated mice deficient for each of the genes. Remarkably, none of the deletion of the individual genes resulted in a detectable phenotypic change. Deletion of all three family members resulted in neonatal lethality within the first few days of age, suggesting that the genes have redundant functions.

## Results

### Identification of a novel IL-2 induced gene

**Figure 1:**
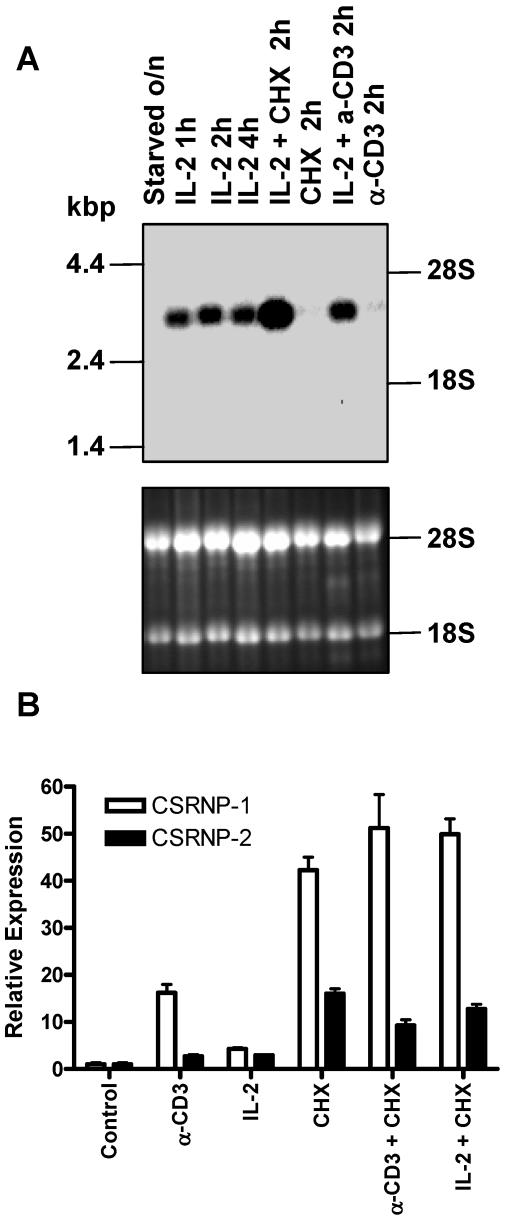
Expression of CSRNP-1 and -2 in T cells. A) Growth arrested activated T cells were stimulated with combinations of IL-2 (200 U/mL), anti-CD3 (0.5 µg/mL) and cycloheximide (20 µg/mL) (CHX) as indicated in the figure, RNA samples were subject to northern blot analysis for CSRNP-1 (upper panel). As a loading control, the gel was stained with ethidium bromide (lower panel) prior to the northern blot analysis B) Lymphocytes from spleen were stimulated with combinations of IL-2 and anti-CD3 for two hours. Total RNA was used to perform quantitative real-time RT-PCR with gene specific primers and probes, to CSRNP-1, -2 and -3. Expression level were calculated from a standard curve made of serially diluted plasmids encoding each genes and normalized to the amount of ribosomal 18S RNA.

To characterize the mechanisms of IL-2-induced T cell proliferation we sought to identify new IL-2 target genes using an Affymetrix array chip (Mu19K). Among the genes that were strongly induced by IL-2 was a new gene that we named cysteine- serine-rich nuclear protein-1 (CSRNP-1). The ortholog of CSRNP-1, AXUD1, was previously identified as a gene that is up-regulated by over-expression of AXIN1 in a human colon cancer cell line [18]. As confirmed by northern blot analysis (Fig. 1A), CSRNP-1 is strongly induced by 1 hour following IL-2 stimulation of activated T cells and expression is maintained for up to 24 hours (data not shown). Inhibition of protein synthesis with cycloheximide did not prevent the induction but rather enhanced it.

### A family of cysteine- and serine-rich nuclear proteins (CSRNP)

**Figure 2:**
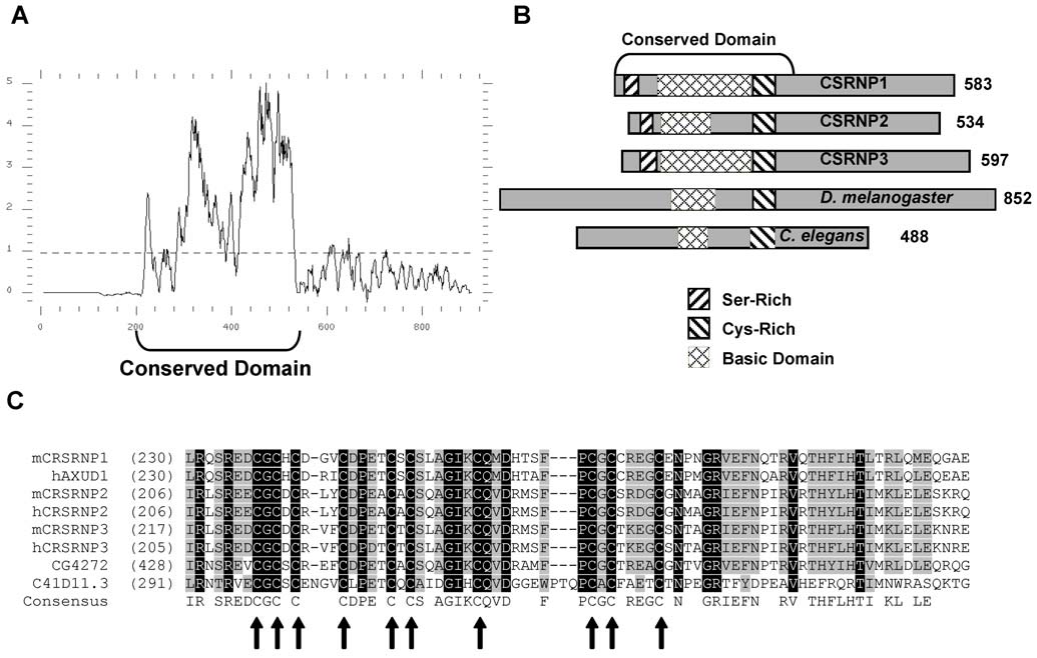
Sequence alignment and primary structure analysis of CSRNP proteins. A) Similarity plot from multiple proteins alignment of CSRNP family proteins. RefSeq from GenBank are as follow: *M. musculus* (m) CSRNP-1 (NP_695019), *H. sapiens* (h) AXUD1 (NP_149016), mCSRNP-2 (NP_700456), hCSRNP-2 (NP_110436), mCSRNP-3 (NP_700458), hCSRNP-3 (NP_079245), *D. melanogaster* CG4272 (NP_608673) and *C. elegans* C41D11.3 (NP_491368). The plot represents the running average of the similarity among the sequences based on a multiple sequence alignment (blosum62 scoring matrix, 10 amino acid comparison windows). The average similarity across the entire alignment is plotted as a dotted line. B) Schematic representation of the primary structures of CSRNP family members with depiction of particular features. C) Multiple sequence alignment of the conserved cysteine (arrows) rich motif.

The predicted structure of CSRNP-1 is illustrated in Fig. 2B. Overall the protein does not contain any previously defined domains. However, the protein contains an amino-terminal region that is serine-rich and a region of approximately 50 amino acids that is cysteine-rich. It should also be noted that overall the protein is serine-rich (15% serine residues). The area between the serine- and cysteine-rich regions contains a predominance of basic amino acids that would have a predicted isoelectric point of 10.5. Based on the overall amino-acid composition, the protein is predicted to have an isoelectric point of 4.6 and to be a nuclear protein (PSORT II program) [19], consistent with the data described below.

A search of the mouse genome database indicated that CSRNP-1 is a member of a family of three related proteins. As illustrated in Fig. 2A, there is sequence similarity across the amino-terminal region of the proteins and includes similarities in the serine- and cysteine-rich domains as well as the basic regions. The most striking similarity was within the cysteine-rich regions (Fig. 2C) which includes 10 positionally conserved cysteine residues. At the protein level, CSRNP-1 is approximately 40% identical to CSRNP-2 and CSRNP-3. Fluorescence *in situ* hybridization (FISH) analysis confirms the sequence maps localization for the CSRNP-1, CSRNP-2 and CSRNP-3 genes to mouse chromosome 9 band F3-4, chromosome 15 band F1-3 and chromosome 2 band C1.3-2 respectively (data not shown). In addition to the mouse genome, searching of other genomic databases identified three CSRNP genes in humans and in most vertebrates, while single genes are present in *D. melanogaster* (CG4272, also termed BcDNA:GH09817) and *C. elegans* (C41D11.3, also termed 1E998 and YK1057). As with the mouse genes, the CSRNP genes of other species were most similar within the cysteine-rich region (Fig. 2C). Since AXUD1 induction is not unique to AXIN1 and, as indicated below, AXUD1 is a member of a structurally related family of proteins, we propose the more descriptive name of cysteine- and serine-rich nuclear proteins (CSRNP) for the gene family.

**Figure 3:**
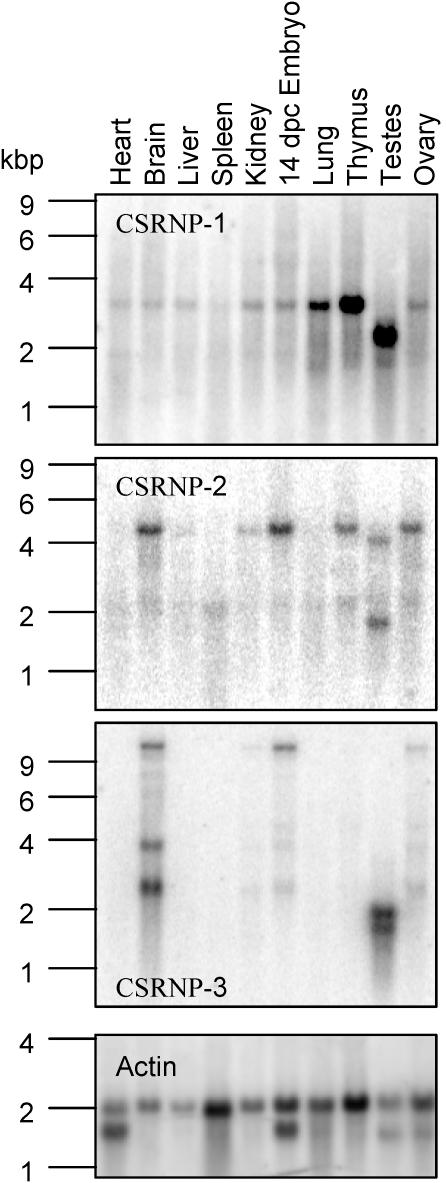
Expression of CSRNP-1, -2 and -3 in mouse tissues. Northern blot analysis of poly(A)+ RNA from multiple murine tissues were hybridized with CSRNP-1, -2 and -3 and actin cDNA probes as indicated on the figure.

Northern blot analyses of the expression in various tissues of the three genes are illustrated in Fig. 3. CSRNP-3 was the most restricted in its expression with expression of a 2.2 kb transcript primarily in the brain with lower levels of expression in kidney and ovary. CSRNP-2 was expressed as a 4.1 kb transcript that was detected in a number of tissues, especially in thymus, brain and ovary. Lastly, a CSRNP-1 transcript of 3.2 kb was detected in all tissues examined. The highest levels of expression were in the thymus and lung. It should be noted that we detected shorter transcripts (of approximate 2 kb) for each gene in testes although the nature of these transcripts was not determined. Since our interest was the potential role of CSRNP-1 in T cells, we also compared the expression levels of the family members in T cells by quantitative RT-PCR. In naive T cells, low levels of transcripts were detected for CSRNP-1 and –2, while CSRNP-3 was undetectable. Moreover, anti-CD3 or IL-2 induced CSRNP-1 transcripts while CSRNP-2 remained unchanged (Fig. 1B).

### CSRNP proteins are potential transcription factors

**Figure 4:**
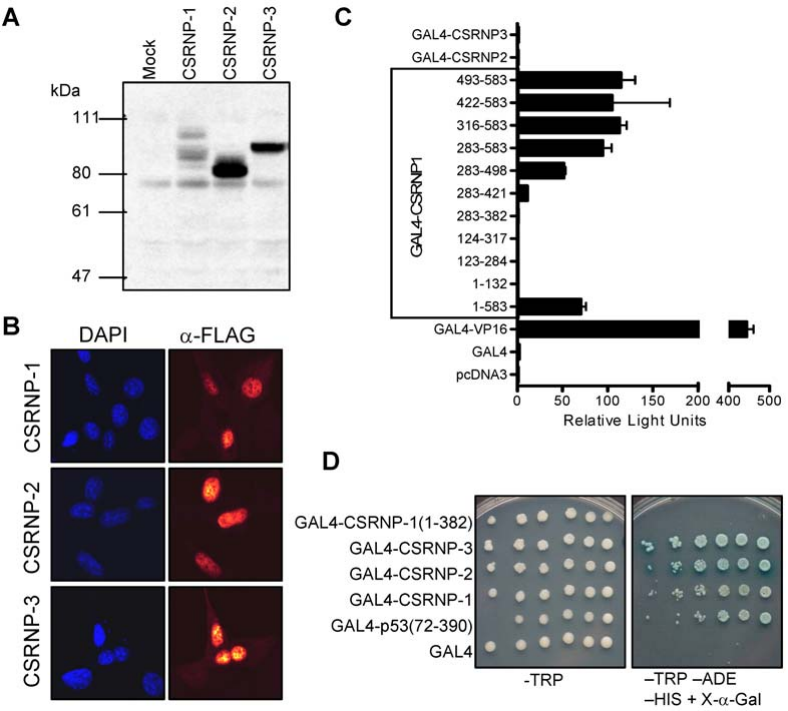
CSRNP proteins localize to the nucleus and activate transcription. A) CSRNP proteins have an apparent molecular weight higher than the predicted molecular weight. Flag-tagged version of CSRNP-1, -2 and -3 were ectopically expressed in 293T cells, proteins were analyzed by western blot with the anti-Flag M2 monoclonal antibodies. B) Immunofluorescence analysis of CSRNP-1, -2 and -3 proteins in NIH-3T3 fibroblasts. CSRNP proteins were by stained with the anti-Flag M2 monoclonal antibodies and visualized with anti-mouse secondary antibody (red). Nuclei were visualized by staining DNA with DAPI (blue). C) Transcription activity of CSRNP proteins in 293T cells. Transcriptional activity of CSRNP-1 was determined by fusing various regions of CSRNP-1 protein to the DNA-binding domain of the yeast GAL4 transcription factor. The constructs were transfected in 293T cells with a GAL4-luciferase reporter and a CMV-β-galactosidase reporter constructs. Luciferase activities were measured and normalized to the the β-galactosidase activities. D) Transcription activity of CSRNP proteins in yeast cells. AH109 cells were transformed as indicated with the CSRNP-1, -2 or -3 GAL4 DNA-binding domain fusions, GAL4p53 (positive control) or GAL4 DNA-binding domain alone (negative control). Transcription activity of the GAL4 fusions was determine by comparative growth assay of serially diluted AH109 transformed cells onto non-selective (left) minimal SD base minus tryptophan (TRP) plate or selective (right) SD minus TRP, minus adenine (ADE), minus histidine (HIS) plus 5-Bromo-4-chloro-3-indolyl-α-D-galactopyranoside (X-α-Gal). The TRP marked plasmid was maintained under selection for tryptophan.

The biochemical properties of the proteins were determined by western blot analysis of Flag-tagged versions of each protein expressed in 293T cells (Fig. 4A). We consistently observed multiple bands for CSRNP-1 with the predominant forms having apparent molecular weights of 90 and 100 kDa. Both of these are larger than the predicted molecular weight (62.5 kDa). In contrast, CSRNP-2 and CSRNP-3 yielded single bands, migrating at apparent molecular weights of 80 and 95 kDa respectively, again larger than the predicted sizes (58.5 kDa and 66 kDa, respectively). The slower than anticipated migration is likely to be due to the acidic nature of the proteins. The Flag-tagged proteins were also expressed into NIH-3T3 cells and analyzed by immunofluorescence staining with the monoclonal M2 anti-Flag antibody. As illustrated (Fig. 4B) the staining of all three family members overlapped with DAPI staining of DNA indicating nuclear localization

Since the CSRNP proteins were nuclear, we sought to determine whether they had transcriptional activity. We designed fusion proteins with the heterologous DNA-binding domain of the yeast GAL4 transcription factor and measured luciferase activity of a GAL4 reporter construct in 293T cells. Among the three full length proteins, only CSRNP-1 stimulated reporter activity (Fig. 4C) that was 70-fold higher than control constructs and 20% of the activity of a fusion protein of the herpes simplex VP16 transactivation domain. Although full length CSRNP-2 and -3 did not have transactivation activity in 293T, similar constructs did transactivate in yeast reporter strains (Fig. 4D). The transactivation domain of CSRNP-1 mapped to the last ninety amino acids (493 to 583) of CSRNP -1 although lower activity was present with fragment containing amino acids 382 and 498.

We then explored the possibility that CSRNP proteins possess DNA-binding activity by using a binding and amplification approach (SELEX) [20], [21]. Table 1 shows the CSRNP-1, -2 and -3 binding sequence determined. For each protein (CSRNP-1, -2 and -3), a non-palindromic sequence of AGAGTG (or the complement CACTCT) was identify at a frequency significantly above random occurrence.

**Table 1:** CSRNP-1, -2 and -3 consensus DNA-binding sequences.

**CSRNP-1, Occured 51/84, E-value = 2.8e-033**						
**Position**	**1**	**2**	**3**	**4**	**5**	**6**
**A**	100%	0%	100%	0%	0%	16%
**C**	0%	0%	0%	0%	0%	22%
**G**	0%	100%	0%	100%	0%	55%
**T**	0%	0%	0%	0%	100%	8%
**Consensus**	**A**	**G**	**A**	**G**	**T**	**G**
**CSRNP-2, Occured 67/67, E-value = 3.6e-060**						
**Position**	**1**	**2**	**3**	**4**	**5**	**6**
**A**	72%	3%	70%	11%	0%	0%
**C**	0%	3%	0%	0%	0%	0%
**G**	1%	67%	29%	89%	48%	91%
**T**	27%	27%	0%	0%	52%	9%
**Consensus**	**A**	**G**	**A**	**G**	**T**	**G**
**CSRNP-3, Occured 64/100, E-value = 1.8e-042**						
**Position**	**1**	**2**	**3**	**4**	**5**	**6**
**A**	100%	0%	95%	0%	0%	6%
**C**	0%	0%	0%	0%	0%	30%
**G**	0%	99%	5%	100%	0%	52%
**T**	0%	0%	0%	0%	100%	13%
**Consensus**	**A**	**G**	**A**	**G**	**T**	**G**

Position-specific matrix of the DNA-binding motif obtained by SELEX.

### Generation of CSRNP-deficient mice

**Figure 5:**
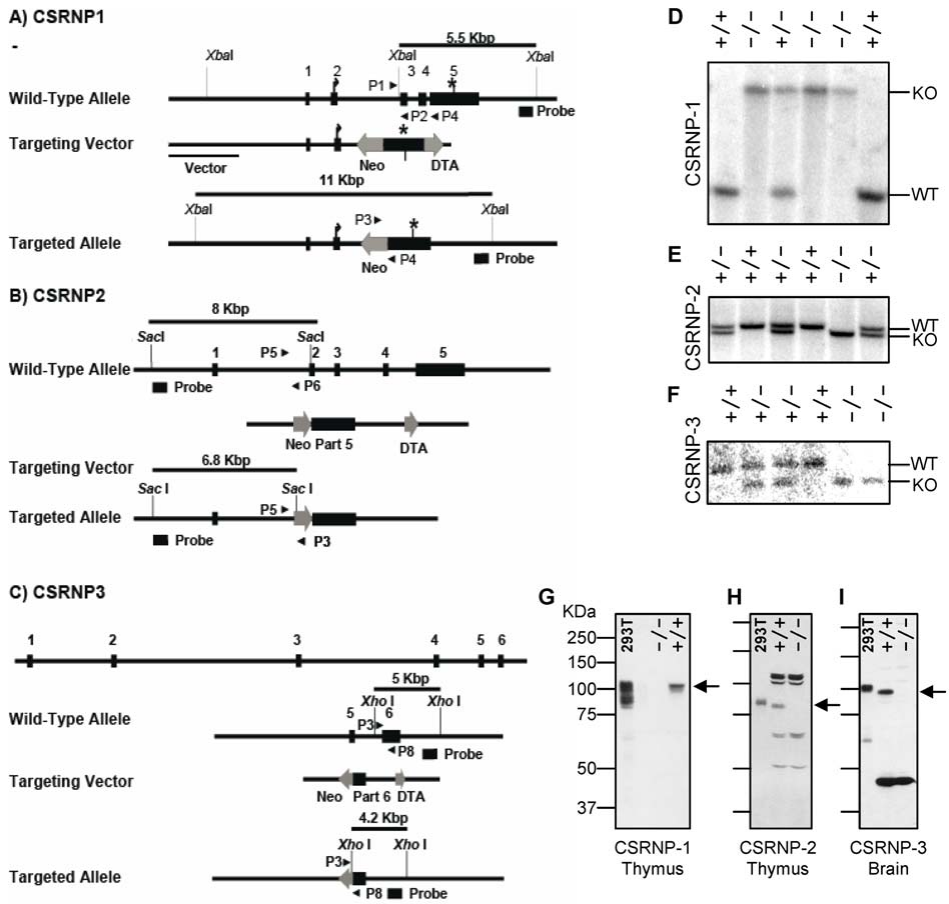
Targeted disruption of CSRNP-1, -2 and -3 in mice. A-C) Schematic representation of the CSRNP-1, -2 or -3 genomic loci and of the targeting constructs. A) CSRNP-1 (GeneID: 215418), B) CSRNP-2 (GeneID: 207785) and C) CSRNP-3 (GeneID: 77771). Neo, neomycin resistance cassette; DTA, diphtheria toxin cassette. Position of the probes used for southern blotting and of the primers (P1 to P8, arrow heads) used for PCR-based genotyping are also shown. D–E) Southern blot analysis of *Xba*I (D), *Sac*I (E) or *Xho*I (F) digested genomic DNA from tail biopsies. Blot was hybridizes with the external probe indicated in (A to C respectively). G–I). Total thymocyte lysates (G–H) or brain homogenates (I) and 293T cells ectopically expressing Flag-tagged version of CSRNP-1, -2 and -3, were separated on a 7.5% SDS-PAGE. Western blot analysis was carried out with affinity purified rabbit polyclonal antibodies raised against a peptides derived from CSRNP-1 (G), CSRNP-2 (H) or CSRNP-3(I). Specific signals are indicated by arrows.

To determine the physiological function of CSRNP proteins *in vivo*, we generated deficient mice for each of the genes. The details of the targeting constructs are described in Materials and Methods and depicted in Fig. 5A–C. All three genes have similar structures and the exon-intron boundaries are at corresponding positions in the proteins. Because of the lack of information about functionally important domains, we sought to delete the most conserved, amino-terminal, portions of the proteins by replacing genomic sequences with a neomycin resistance cassette. Because the CSRNP-3 gene spanned over 180 Kbp, a smaller deletion was used. In all cases the conserved cysteine-rich domain was deleted. Proper targeting and transmission of the allele was confirmed by Southern blot analysis (Fig 5D–F). To determine if the targeting strategies produced null alleles, we raised antisera against peptides derived from the CRSNP-1, -2 and -3 protein sequences. Affinity purified antibodies were used to probe western blots of lysates from wild type and CSRNP deficient tissues. In each case, specific bands were detected in wild type tissues but not in tissues from each deficient strain, again the endogenous proteins migrated much slower than their predicted molecular weight but slightly faster than the corresponding flag-tagged versions owning to the presence of the tag (Fig. 5G–I). We thus conclude that the targeting in each mutant strain produced a functionally null allele.

### Analysis of CSRNP deficient mice

**Figure 6:**
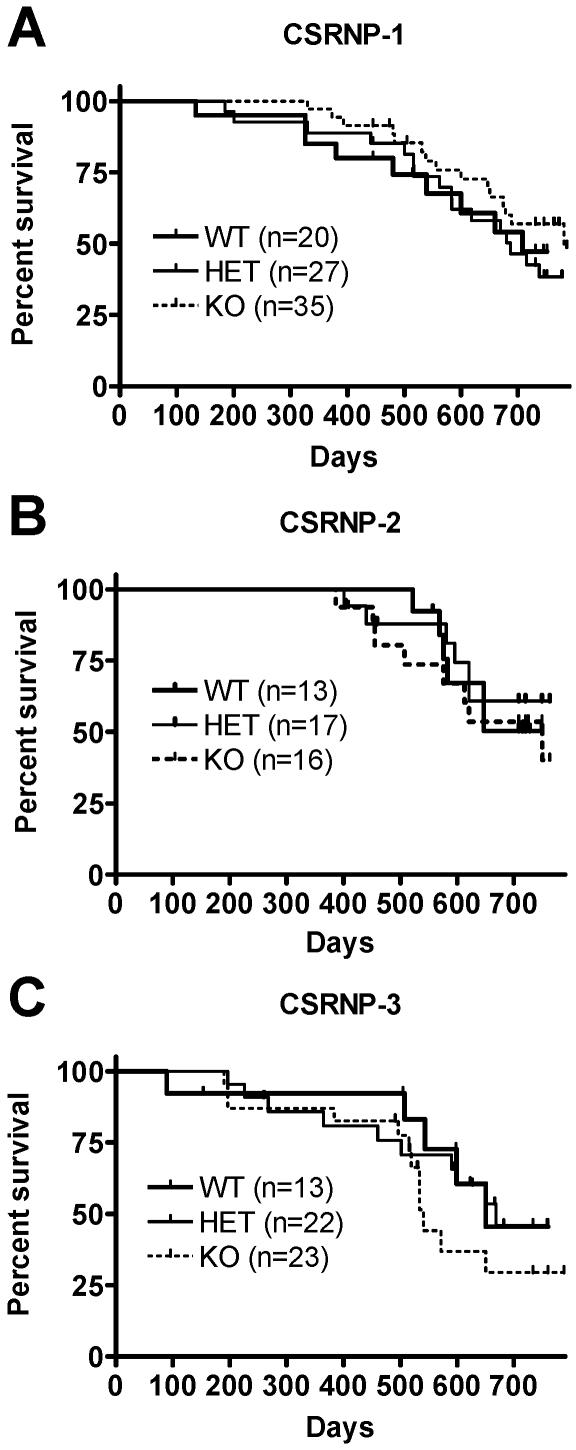
The longevity of the CSRNP-deficient mice was similar to the heterozygous and wild type control mice. Kaplan-Meier survival curves of CSRNP-1 (A), CSRNP-2 (B) and CSRNP-3 (C) littermates mice, wild type (WT) heterozygous (HET) and homozygous (KO). Curves are not significantly different, p value >0.5.

Interbreeding of heterozygote mice produced litters containing mutant mice at the expected Mendelian ratio. CSRNP-1, 2- or 3-deficient mice were indistinguishable from their wild type and heterozygote littermates, developed normally and appeared healthy. Male and female deficient mice are fertile, and females appropriately nursed their offspring to adulthood. Histological examination of various organs from CSRNP-deficient mice did not reveal any abnormalities. Cohorts of deficient mice were followed for over two years without evidence of increased death rates (Fig. 6A–C) and there were no differences in the spectrum of pathologies observed.

**Figure 7:**
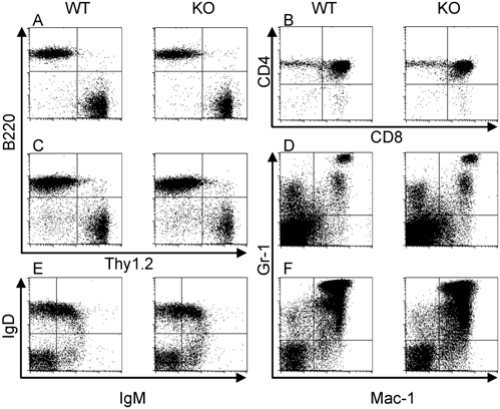
Flow cytometric analyses of hematopoietic cells. Lymph node lymphocytes (A), thymocytes (B), splenocytes (C and D) or bone marrow cells (E and F) from individual wild type and CSRNP-1 knockout mice were stained with monoclonal antibodies to B220 and Thy1.2 (A and C), CD4 and CD8 (B), Mac-1 and Gr-1 (D and F) or IgM and IgD (E). Data presented are from a pair of littermate representative of more than 5 pairs analyzed.

The cellularities of hematopoietic tissues (bone marrow, thymus, spleen, and lymph nodes) of CSRNP-1 deficient mice were comparable to wild type littermates (data not shown). Flow cytometry analysis of mature lymphocytes from lymph nodes (Fig. 7A) and spleen (Fig. 7C) with anti-Thy1.2 and anti-B220 revealed no differences in the number of T- and B cells. Analysis of developing thymocytes revealed no difference in the proportions of double-negative (CD4- CD8-), double-positive (CD4+ CD8+), and single-positive (CD4+ CD8- or CD4-CD8+) cells (Fig. 7B), nor did we find any difference at any stage of double-negative development as measured by staining with anti-CD25 and anti-CD44, (data not shown) indicating that T cell development is unaffected by CSRNP-1-deficiency. The numbers of immature B cells in the bone marrow as measured by anti-IgM and anti-IgD staining of B220+ cells (Fig. 7E) or by staining with anti-B220 and anti-CD43 of IgM- cells (data not shown) were also normal. Analysis of the myeloid compartment with anti-Mac-1 and anti-Gr-1 did not reveal any effect of CSRNP-1 deficiency in neither spleen (Fig. 7D) nor bone marrow cells (Fig. 7F). Similarly, the numbers of Ter119, c-kit, or NK1.1 cells were normal in CSRNP-1-deficient mice (data not shown). Taken together those results indicate that CSRNP-1 is not essential for normal mouse hematopoiesis. Similar analyses were performed with CSRNP-2 and -3 deficient mice, as well as CSRNP-1/-2 double knockouts, and in every case we failed to observe any impact of CSRNP deficiency on hematopoiesis.

**Figure 8:**
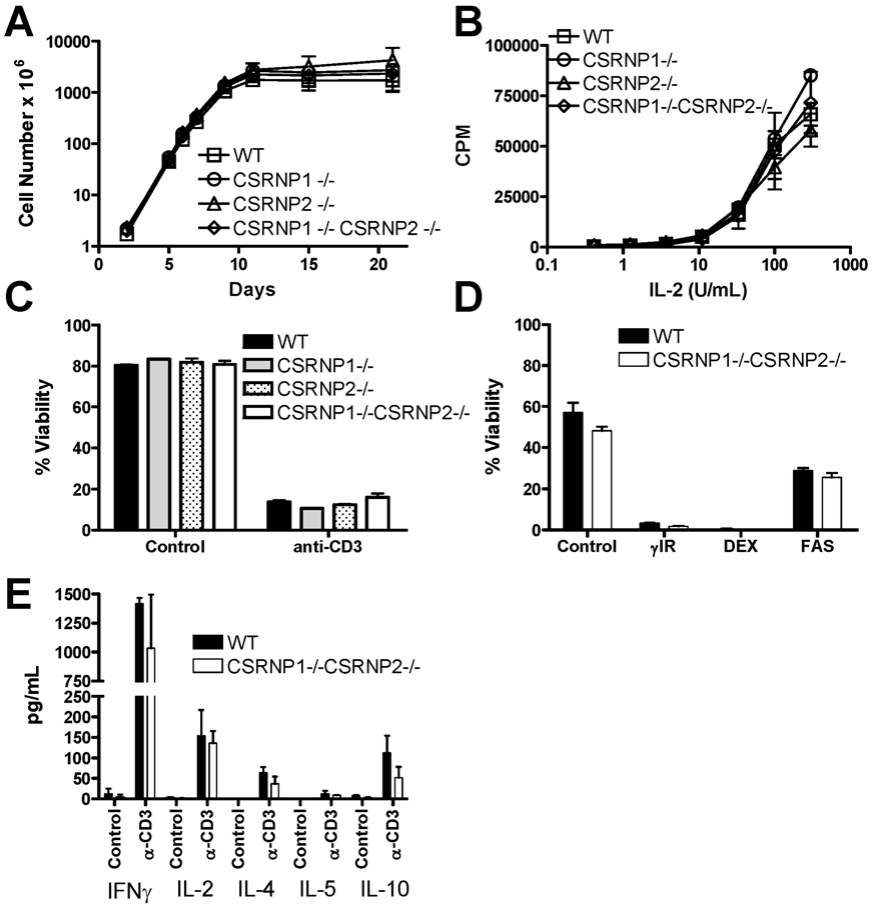
Normal T cells functions in CSRNP-deficient mice. A) Growth curves of wild type, CSRNP-1^−/−^, CSRNP-2^−/−^ and CSRNP-1^−/−^/CSRNP-2^−/−^ T cells. Splenocytes were seeded a density of 10^6^ cells/ml and stimulated with anti-CD3 and IL-2, thereafter, the cultures were kept in IL-2-containing media. Viable cell counts were determined by trypan blue staining every 2–3 days and the cells were split back to 10^6^ cells/ml. The data shown represent the average of three animals per genotype. B) Activated T cells grown in the presence of IL-2 for 7 days were IL-2-depreived overnight and then stimulated with increasing amounts of IL-2 for 48 hours, then DNA synthesis was determined by pulsing with [^3^H]thymidine for 16 hours. C) Activated T cells grown in the presence of IL-2 for 5 days were then stimulated overnight with plate-bound anti-CD3, viability was determined by trypan blue staining. D) Thymocytes were either gamma-irradiated (IR) with 500 rad or treated with 1 µM of dexamethasone (DEX) or 1 µg/mL anti-FAS, 20 hours later viability was determined by propidium iodine exclusion using flow cytometry. E) Cytokine secretion by activated T cells. T cells were purified by MACS and left unstimulated or treated with plate-bound anti-CD3 for 2 days. Thereafter, supernatant were harvested and cytokine secreted in the media were measured using Bio-Plex technology. There were no significant difference (p>0.05) between any genotypes in any of these graphs.

Since CSRNP-1 is strongly induced by IL-2 in activated T cells, we examined the ability of CSRNP-1-deficient T cells to proliferate in response to IL-2. Because, we also detected transcript for CSRNP-2 in T cells, and because these genes could be redundant, we also analyzed CSRNP-2^−/−^ and CSRNP-1^−/−^/CSRNP-2^−/−^ mice. Splenocytes were activated with anti-CD3 and IL-2 and cultured in media containing IL-2 alone. Irrespective of their genotype, activated T cells proliferated in response to IL-2 for 10 days (Fig 8A). To determine if CSRNP-1 and/or -2 deficiency affect the sensitivity of activated T cells to response to IL-2, proliferation in response to increasing concentrations of IL-2 was measured by thymidine incorporation. The proliferation of wild type and knockout T cells was undistinguishable at any concentration of IL-2 (Fig. 8B).

IL-2 has been shown to promote AICD of T cell upon TCR re-stimulation [6], so we examined if CSRNP-1 deficiency had any impact on AICD. Activated T cells were grown in IL-2 for five days and then restimulated with plate-bound anti-CD3 in the presence of IL-2. Under those conditions, we found that knockout activated T cells were as susceptible as wild type to apoptosis, similarly IL-2 withdrawal also induced cell death to the same extent in both genotypes (Fig. 8C).

AXIN1, an inducer of CSRNP-1/AXUD1, induces a high level of apoptosis in spleen and thymus when over-expressed as a transgene [22]. Since both CSRNP-1 and -2 are constitutively expressed in thymocytes (Fig. 3), we investigated whether apoptosis of thymocytes was altered in CSRNP-1/-2 double knockouts. Wild type and CSRNP-1^−/−^/CSRNP-2^−/−^ thymocytes were cultured with dexamethasone, agonistic anti-Fas antibody or received γ-irradiation. Dexamethasone and γ-irradiation completely killed double positive thymocytes while anti-Fas antibody reduced viability by roughly 50% but there was no significant difference between wild type and CSRNP-1^−/−^/CSRNP-2^−/−^ thymocytes (Fig. 8D). Similar results were obtained with single CSRNP-1^−/−^ or CSRNP-2^−/−^ thymocytes (data not shown).

One of the main functions of T cells upon activation is to produce cytokines, MACS-purified T cells were stimulated with plate-bounded anti-CD3 for 48 hours then supernatant where harvested and a few critical cytokines were quantified by multiplex beads assay (Fig. 8E). Again, there were no differences between wild type and CSRNP-1^−/−^/CSRNP-2^−/−^ double knockout T cells in the level of IFNγ, IL-2, -4, -5 and -10 secreted.

### CSRNP have potential redundant functions for perinatal viability

**Figure 9:**
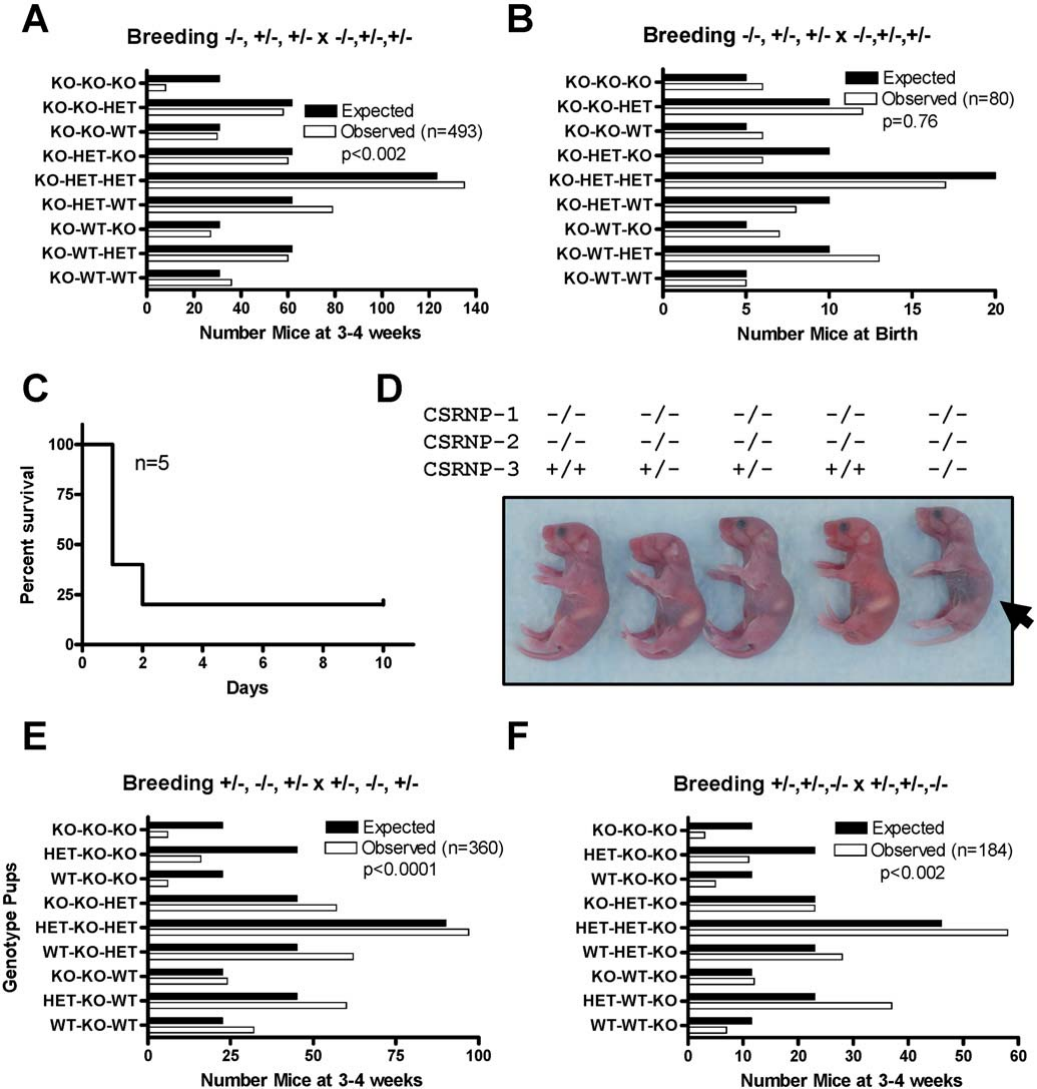
CSRNP-1, -2 and -3 genes have redundant function required for mice survival. Comparison of the observed distribution of genotypes to the expected frequency for offspring obtained from CSRNP-1^−/−^/CSRNP-2^+/−^/CSRNP-3^+/−^ breeder pairs at A) 4 weeks and B) birth. C) Kaplan-Meier survival curves of CSRNP-1^−/−^/CSRNP-2^−/−^/CSRNP-3^−/−^ triple knockouts. D) Normal morphological phenotype of CSRNP-1^−/−^/CSRNP-2^−/−^/CSRNP-3^−/−^ triple knockout newborn with the exception of the absence of milk in their stomach. E–F) Comparison of the observed distribution of genotypes to the expected frequency for offspring obtained from CSRNP-1+/−/CSRNP-2−/−/CSRNP-3+/− and CSRNP-1+/−/CSRNP-2+/−/CSRNP-3−/− breeder pairs at 4 weeks, respectively. Chi square test were performed to determine the significance of differences between the expected and observed distributions.

To determine whether the CSRNP genes have redundant function, we analyzed the offspring, at 3–4 weeks of age, from the mating of CSRNP-1^+/−^, CSRNP-2^+/−^, CSRNP-3^−/− ^mice (Fig. 9A). Mice deficient for all three genes were only obtained at 25% of the expected frequencies. Genotyping pups right after birth, demonstrated that triple knockouts were born at the expected frequency (Fig. 9B), but the majority died during the first 48 hours (Fig. 9C), for reasons that were not evident from overt or histological analysis with the possible exception of lack of milk uptake (Fig. 9D). Analysis of surviving CSRNP-1, -2 and -3 triple knockouts, indicated that hematopoiesis was not affected (data not shown). To determine whether CSRNP-1 contributed to the phenotypes we also analyzed the offspring from CSRNP-1^+/−^, CSRNP-2^−/−^, CSRNP-3^+/−^ (fig. 9E) and CSRNP-1^+/−^, CSRNP-2^+/−^, CSRNP-3^−/−^ (fig. 9F) and again, triple knockouts were obtained at 25% of the expected frequencies. In addition, the number of observed offspring relative to that expected of CSRNP-2^−/−^, CSRNP-3^−/−^, irrespective of the whether they were CSRNP-1^+/+^ or CSRNP-1^+/−^, ranged from 25% to 45%, indicating that deficiency in CSRNP-2 and CSRNP-3 results in a significant reduction of survival. There is no significant difference between the survival of CSRNP-2^−/−^, CSRNP-3^−/−^ and that of the triple mutants, therefore CSRNP-1 is likely to not contribute to the phenotype.

## Discussion

Gene array analysis has been widely used to characterize the changes in gene transcription in cellular responses. This global approach to gene expression has the advantage of identifying novel genes potentially involved in the cellular responses. Using this approach several studies identified AXUD1/CSRNP-1 as differentially regulated. Specifically, it is induced with the over-expression of AXIN1 potentially implicating the Wnt signaling pathway. It was identified in a comparative genomics array approach as an immune function associated gene [23], a PDGF transcriptional target by microarray-coupled gene-trap mutagenesis approach [24], an inducible gene in microglial cells [25], a doxorubicin induced gene in cardiomyocytes [26] and, as demonstrated here, an IL-2 induced gene. In addition, the sequences for all three genes have been deposited in GenBank as TGF-β induced apoptosis proteins (TAIP). Conversely, AXUD1/CSRNP-1 has been shown to be down-regulated in microarray studies in a variety of tumors including lung, liver, colon and kidney cancers [18], intrahepatic cholangiocarcinomas [27], pancreatic tumors [28] and breast cancers [29]. Given the wide range of settings in which the AXUD1/CSRNP-1 gene expression is either up- or down-regulated, it was important to gain further insights into the potential physiological functions of the gene.

Beyond the wide range of factors that affect AXUD1/CSRNP-1 expression, the properties of the gene product and its family members make the CSRNP family of proteins interesting. All are nuclear proteins with considerable similarity in their DNA sequence preference for binding to DNA. Although, we have not defined the domains of the protein that are involved in the DNA binding activity, the similarity of sequences observed with all three family members would suggest that it involves a conserved region, possibly the cysteine-rich or the basic domain.

One of the more definitive approaches to defining gene function is through the derivation of mouse strains in which the genes are deleted. However, in the case of the CSRNP family of genes, derivation of null mutants in mice failed to reveal any obvious changes in phenotype that could be used to further study function. Similarly, there is a reported knockdown of the *C. elegans* gene by RNA interference that resulted in no obvious phenotype (http://www.wormbase.org, WBGene00016562) as well as a report of a P element insertion in the promoter in *D. melanogaster* that does not affect viability (http://flybase.org, allele CG4272^KG07402^). Based on the strong induction of CSRNP-1 in peripheral T cells in response to IL-2, we focused primarily on mice deficient in this gene. As detailed, there were no changes in T cell development, the ability to proliferate in response to IL-2, in cytokine production or in the ability to undergo antigen-induced cell death. We also examined the ability of the CSRNP-1 deficient mice to respond to influenza virus infections without observing any changes (data not shown). Although not induced by IL-2, CSRNP-2 is also expressed at low levels in peripheral T cells and consequently similar studies have been done with CSRNP-2 deficient and CSRNP-1/-2 double deficient mice to explore potential functional redundancy. Preliminary studies indicate that CSRNP-2 deficient mice were more susceptible to influenza infection (data not shown), but it appears that it does not affect the generation of influenza virus-specific T cells. The increased susceptibility is still under investigation. A mutant allele of AXUD1/CSRNP-1, generated by gene trapping was recently reported [30], it is associated with a 20% post-natal lethality and a weakly penetrant reduction of PDGFRα-dependent skeletal structures. We have analyzed over 300 offspring at weaning from interbreeding of heterozygote CSRNP-1 mice and obtained exact mendelian ratios and no evidence of post-natal lethality. We also did not observed skeletal defects, therefore our knockout does not support the phenotypes reported.

Deficiencies in both CSRNP-2/-3 or in all family members produced a partial neonatal lethality within the first 48 hours of birth. However we have been unable to determine the basis for the lethality but it may be associated with a reduced vigor or failure to compete for material support. Histological analysis failed to identify phenotypic differences consequently the basis for the partial neonatal lethality is unknown. Therefore the contribution of CSRNP-1-/- to the phenotype will only be definitively addressed once the mechanisms of action of the CSRNP proteins are identified. Also crossing the mutant mice on pure strains might increase the penetrance of this phenotype and help to identify the defect.

The lack of any phenotypic consequences of deleting CSRNP-1, CSRNP-2 or both genes in T cells can be interpreted in several ways. One possibility is that the genes have critical functions, but their functions are redundant. Normally redundancy is considered between family members, however in the case of CSRNP-1 our studies clearly demonstrate that, if redundancy is the basis for lack of any consequences of deleting the genes on T cells, the redundancy must be with gene(s) unrelated to the CSRNP family of genes. Alternatively, the CSRNP genes may be essential for a cellular response to a specific set of conditions or factors.

A number of studies have identified AXUD1/CSRNP-1 as a gene that is down-regulated in a variety of cancer, thus implicating the gene as a potential tumor suppressor gene, often considered related to reduced apoptosis. However, deficiencies of the individual family members failed to show any increase in cancers. Moreover, in studies of T cells and hematopoietic cells, we did not detect any reduction in apoptosis as a consequence of deficiencies of the genes for the individual family members. Irrespective, as global approaches to gene expression continue to implicate members of the CSRNP family of genes in various physiological responses, the availability of strains of mice lacking the genes will be invaluable to critically assess their physiological role.

## Materials and Methods

### T cells assays

Spleens, thymi, or lymph nodes were crushed into single cell suspensions in PBS containing 2% fetal bovin serum (FBS) through a 70-µm-pore-size nylon mesh. Bone marrow cells were obtained by flushing femurs and tibias with the same buffer. Red blood cells were lysed by addition of buffer (pH 7.2) containing 150 mM NH_4_Cl, 1 mM KHCO_3_, and 0.1 mM EDTA; debris were removed by straining. To activate T cells, single-cell suspensions (10^6^ cells/ml) of naïve lymphocytes from spleen or lymph nodes were stimulated with 0.5 µg/ml of anti-CD3 (2C11; BD Biosciences Pharmingen, San Diego, CA) and 200 U/ml of recombinant human IL-2 (Chiron Corp. Emeryville, CA) in RPMI 1640 media supplemented with 10% FBS, L-glutamine, penicillin, streptomycin, sodium pyruvate, nonessential amino acids (all from Life Technologies), and β-mercaptoethanol (Sigma). To determine the growth curve of T cells, cells were kept in IL-2-containing media, counted every second day and were split back to 10^6^ cells/ml. Viable cell counts were determined by trypan blue exclusion on Vi Cell™ Beckman Coulter Counter.

For some experiments activated T cells were growth arrested by overnight IL-2 withdrawal. To assess the proliferative response to IL-2, 2×10^5^ cells growth-arrested T cells were plated in triplicate in individual well of a 96-well round-bottomed plate in 200 µl of media with indicated amounts of IL-2. After 48 h of stimulation, 1 µCi of [3H]thymidine (PerkinElmer Life Sciences Inc., Boston, MA) was added to the culture for another 16 h. Cells were harvested and incorporated [3H]thymidine was measured as counts per minute 1450 MicroBeta® Trilux liquid scintillation detector (PerkinElmer Life Sciences Inc.).

For fluorescence-activated cell sorter (FACS) analysis, cells were labeled with fluorophor-conjugated monoclonal antibodies (all from BD Biosciences Pharmingen) directed against Thy1.2 (53-2.1), B220 (RA3-6B2), CD3 (17A2), CD4 (L3T4), CD8 (53-6.7), IgM (R6-60.2), IgD (11_26C.2X), NK1.1 (PK136), TCRβ (H57-597), Gr-1 (RB6-8C5) CD11b (Mac-1, M1/70) and Ter119. The stained cells were then analyzed on a FACSCalibur (Becton Dickinson) in multi-color mode using CellQuest software.

Pure T cells (>95%) were obtained by negative selection using magnetic cell sorting. First, splenocytes were labeled with PE-conjugated antibodies (utilizing a cocktail of anti-B220, anti-Gr-1, anti-Mac-1, anti-NK1.1 and anti-Ter119). The cells were then magnetically labeled with anti-PE MicroBeads (Miltenyi Biotech, Auburn, CA) according to manufacturer's instructions, before being loaded on an AutoMACs (Miltenyi Biotech) for the depletion of labeled cells.

Cytokines were measured on a Bio-Plex™ (Bio-Rad Laboratories Inc., Hercules, CA) following manufacture's instructions, using Luminex beads (MiraiBio Inc., CA), and ELISA matched antibodies pairs (BD Biosciences Pharmingen).

### RNA analysis

We compared gene expression of wild type activated T cells (99% Thy1.2+ and 85% CD8+) that were deprived of IL-2 for 16 h to activated T cells that were starved and then re-stimulated with IL-2 for 2 hours. Total RNA was extracted with TRIzol® reagent according to the manufacturer's instructions (Invitrogen Corp., Carlsbad, CA). We performed a screen using the GeneChip® Mu19K array (Affymetrix, Santa Clara, CA) (unpublished data). The genes on this array were based on sequences from The Institute for Genomic Research (TIGR) databanks (www.tigr.org).

For Northern blot analysis, total RNA was separated by formaldehyde gel electrophoresis, and transferred to Hybond-N+ positively charged nylon membrane (Amersham Biosciences Corp., Piscataway, NJ). The various probes were labeled with the Rediprime™ II kit (Amersham Biosciences Corp.) and hybridization was performed in Rapid-hyb buffer (Amersham Biosciences Corp) following manufacturer's instructions. A FirstChoice™ Northern blot of murine poly(A) RNA (Ambion Inc., Austin, TX) was sequentially probed with radiolabeled fragment of CSRNP-1, -2, -3 and β-actin cDNA.

Real time RT-PCR was performed, using TaqMan® EZ RT-PCR reagents (Applied Biosystems, Foster city, CA) following supplier's instructions on an ABI Prism™7700 sequence detector (Applied Biosystems). The following forward primer, reverse primer and probe (in this order) were used for CSRNP1: TCCGCCGCCGTTTAAAG, ATCGATGGCATCCGCTGT, FAM- CGGAACTCCAGCCACTGAAAACTTCCA-TAMRA; for CSRNP2: GGAGGAGAAACTTCACGCCA, ATCGAGTGTCAAGCCGTCG, FAM- CGGTCCCGTTCTTCGTCAGCTTCA-TAMRA; for CSRNP3: GATGCTGAGAGAACACCTCCG, CCACTTCGGTGTTGTCCAGA and FAM-ACCCTGACCGTGGATGACATTTCCG-TAMRA. All amplifications were performed in duplicate with 100 ng of total RNA. A standard curves consisting of serial dilutions from 10^1^ to 10^6^ copy of plasmids containing each cDNAs were included in every assays. The determined copy number of CSRNP mRNA was normalized to the amount of 18S rRNA in each sample (TaqMan® Ribosomal RNA control reagents (Applied Biosystems).

### Cloning of CSRNP cDNAs

We identified one highly regulated probe set corresponding to TIGR contig accession number TC39688. We used a corresponding EST fragment (IMAGE clones no. 466039, GenBank accession no. AA031119) as a probe to screen a mouse spleen 5′-Stretch Plus cDNA library, (Clonetech Laboratories Inc., Palo Alto, CA). One positive clone of 2570 bp was isolated, sequence analysis revealed that it contained a poly A tail and an open reading frame of 1488 bp, which was latter found to encode amino acids 89 to 583). 5′-RACE was performed using Marathon cDNA Amplification Kit (Clonetech Laboratories Inc.) on poly A+ mRNA isolated from activated T cells to generate a full-length cDNA. Our sequence is essentially the same as the GenBank RefSeq NM_153287. We first identified CSRNP-2 by a related mouse EST sequence (BF122209) the IMAGE clone was ordered from Incyte and sequenced. The complete sequence (4168 bp) was since deposited in GenBank (BC03595) by the Mammalian Gene Collection Program Team. We identified CSRNP-3 genomic sequence corresponding to exon 4 and 5 from a BAC clone. This sequence was used to design primer to amplify a full cDNA from a mouse liver library by 5′- and 3′-RACE, the sequence obtained is essentially the same as the GenBank RefSeq NM_153409.

### Chromosomal localization

CSRNP-1 –2 and -3 genomic clones were isolated from a W9.5 embryonic stem cell BAC library (Incyte Genomics, St. Louis, MO) by screening with cDNA probes. BAC clones were identified and used for chromosomal localization of each gene by fluorescence in situ hybridization (FISH). The DNA was labeled with digoxigenin-11-dUTP (Roche Molecular Biochemicals, Indianapolis, IN), and hybridization was performed essentially as previously described [31] on metaphase chromosomes derived from early passage mouse embryonic fibroblasts.

### CSRNP targeting vector and generation of mutant mice

For the CSRNP-1 gene disruption targeting vector, a 9.5 Kbp *Xho*I-*Apa*I genomic fragment was isolated from a BAC clone and used to construct the targeting vector. A 3.2 Kbp *Eco*RI fragment containing exon 3, 4 and the first 72 bp of exon 5 was replaced with a cDNA encoding the neomycin resistance gene driven by the thymidine kinase promoter oriented in the direction opposite to that of CSRNP-1 transcription. For the CSRNP-2 gene disruption targeting vector, a 2.9 Kbp *Hin*dIII-*Bgl*II upstream of exon 2 was subcloned upstream of the neo cassette, and a 5.8 Kbp *Xho*I containing part of exon 5 was cloned downstream, in effect creating a targeting vector, where exons 2, 3, 4 and the first 290 bp of exon 5 are deleted. For the CSRNP-3 gene disruption targeting vector, a 9 Kbp *Bam*HI genomic fragment was isolated from a BAC clone and used to construct the targeting vector. A 3.8 Kbp *Xba*I-*Apa*I fragment containing exon 5 and the first 203 bp of exon 5 was replaced with a cDNA encoding the neomycin resistance gene driven by the thymidine kinase promoter oriented in the opposite direction to that of CSRNP-3 transcription. For all three targeting vector, a cDNA encoding the diphtheria toxin gene [32] driven by the thymidine kinase promoter was inserted at the 3′of the targeting construct and was used for negative selection. CSRNP-deficient mice were generated essentially as previously described [33]. Briefly, 129/SvJ E14 ES cells were electroporated with 20 µg of linearized targeting vector and selected with 340 µg/ml of geneticin (Invitrogen Corp). Southern Blot analysis was performed to identify clones with proper integration of the targeted allele. DNA from geneticin-resistant clones was digested with *Xba*I for CSRNP-1 clones, *Sac*I for CSRNP-2 clones or *Xho*I for CSRNP3 clones; and then probed with labeled DNA fragment adjacent to the targeted region. Clones heterozygous for the mutation were injected into C57BL/6 blastocyts, which were then implanted into pseudopregnant females. Heterozygote agouti mice, from chimeras passing the mutation through the germ line, were interbred to produce wild type and CSRNP-deficient littermates on a mixed 129/SvJ/C57BL/6 background. Genotype was routinely determined from tail DNA by PCR using the following primers for CSRNP-1: wild type allele, P1 (CACCGTCTACTATTTCCCACGGTG) and P2 (TACCTTCCACCTCAGCATCTCCAG) (202 bp) and knockout allele P3 (CTGCGTGCAATCCATCTTGT) and P4, (GAAGTGCGTCTGAACTCTTGTCTG) (378 bp); for CSRNP-2: wild type allele, P5 (GGAGGCTGATGAGACACTTC) and P6 (AGGAGAGCACTCCAAGGTAT) (452 bp) and knockout allele P3 and P5 (375 bp); for CSRNP-3: wild type allele, P7 (CCTTCCAAGGTGGATCGTATGT) and P8 (TGGCAGTCCAGTTCCTCCTGTA) (314 bp) and knockout allele P3 and P8 (431 bp).

For organ histological analysis, tissues were fixed overnight in 10% buffered formalin (Fisher Scientific). Fixed tissues were embedded in paraffin, sectioned (10 µm), and stained with hematoxylin and eosin.

### Transfections, western blots and immunofluoresence

NIH-3T3 and 293T cell were transfected using the classical calcium phosphate methods. Cell lysates were prepared in Laemmli sample buffer and separated by SDS-PAGE. Proteins were transferred to nitrocellulose membranes, blocked with 3% dry milk in TBST (10 mM Tris [pH 8.0], 150 mM NaCl, 0.1% Tween 20), incubated at room temperature for 1 h with anti-FLAG® M2 (Sigma Chemical Co., St-Louis, MO), washed with TBST, incubated for 1 h with sheep anti-mouse Ig horseradish peroxidase-conjugated antibody (Amersham Biosciences Corp), and proteins were visualized with ECL™ western blotting detections reagents according to the manufacturer's instructions (Amersham Biosciences Corp).

Immunofluorescence was performed on NIH-3T3 transfected with FLAG tagged versions of the cDNAs. Cells were first fixed to glass slides for 20 min at −20°C in methanol:acetone (1:1), then pearmibilized with 0.1% Triton-X100 for 5 min at room temperature. Slides were then stained with anti-FLAG® M2 and detected with Alexa Fluor® 568 goat anti-mouse antibody (Molecular Probes Inc, Eugene, OR).

Analysis of transactivation activity was performed as previously described [34]. Briefly, a series of fusion proteins were generated by standard molecular biology techniques between CSRNP proteins and the heterologous GAL4 DNA-binding domain. The plasmids encoding the fusion proteins were co-transfected with a control reporter vector CMV-β-galactosidase and a luciferase reporter construct under the control of GAL4 response elements. β-galactosidase activity was determined with a chemiluminescent reporter assay kit (Galacto-Light Plus™, Tropix, Bredford, MA), luciferase activity was measured with luciferase assay system (Promega, Madison, WI) and normalized to the β-galactosidase activity, the normalized luciferase values are given as arbitrary units.

Analysis of transactivation activity in yeast was performed with the MATCHMAKER Two-Hybrid System (Clontech) according to manufacture's instructions. CSRNP-1, -2 and -3 were cloned in frame with GAL4 DNA-binding domain in pGBKT7 cloning vector. The plasmids were transformed in the yeast strain AH109 bearing the HIS3, ADE2 and LacZ reporter genes.

### Systematic evolution of ligands by exponential enrichment (SELEX)

SELEX was performed essentially as previously described [20]. Briefly, Flag-tagged versions of each CSRNP proteins were transfected in 293T cells. For each binding reaction the Flag-tagged proteins from one 10 cm dish was immunoprecipitated with Anti-FLAG M2 affinity gel. The immunocomplex was incubated with an unbiased set of totally degenerate 30 bp sequence flanked by PCR priming sequences. The random double-stranded oligonucleotide was: 5′-CGCGGATCCTGCAGCTCGAGN1-N_30_-GTCGACAAGCTTCTAGAGCA-3′. The primers for the PCR were as follows: forward, 5′-CGCGGATCCTGCAGCTCGAG-3′; reverse, 5′- TGCTCTAGAAGCTTGTCGAC-3. The bound DNA was amplified by PCR and the PCR-product used again in a subsequent binding reaction. Specific binding was obtained after 4 to 6 cycles; selected binding sites were then cloned into pCR2.1-TOPO vector (Invitrogen) and sequenced. Sequences trimmed of primers and vector sequences were used as input for the motif discovery tools MEME which outputs all motifs present in the training set.

### Rabbit polyclonal antibodies

Peptides corresponding to CSRNP-1 amino acids 36 to 54 (ASSASPAWNSDEEGPGGQA), CSRNP-2 amino acids 484 to 502 (KEPESEDLHPSWSPSSLPF) AND CSRNP-3 amino acids 561 to 581 (GDSHISEHPAENPLSLAEKSR) were synthesized, conjugated to glutaraldehyde-activated keyhole limpet hemocyanin, and used to immunize rabbits (Rockland Immunochemicals, Inc. Gilbertsville, PA). Before use, antibodies were purified by affinity chromatography over corresponding peptide columns.
